# Yeast Fermentate Prebiotic Ameliorates Allergic Asthma, Associating with Inhibiting Inflammation and Reducing Oxidative Stress Level through Suppressing Autophagy

**DOI:** 10.1155/2021/4080935

**Published:** 2021-01-19

**Authors:** Subo Gong, Xiaoying Ji, Jing Su, Yina Wang, Xianghong Yan, Guyi Wang, Bin Xiao, Haiyun Dong, Xudong Xiang, Shaokun Liu

**Affiliations:** ^1^Second Xiangya Hospital of Central South University, No. 139 Middle Renmin Road, Changsha City, Hunan Province, China; ^2^Pulmonary and Critical Care Medicine, The Seventh Affiliated Hospital, Sun Yat-sen University, No. 628, Zhenyuan Road, Xinhu Street, Guangming District, Shenzhen City, China; ^3^Department of Laboratory Medicine, Changsha Central Hospital, Nanhua University, Changsha City, Hunan Province, China

## Abstract

**Methods:**

Ovalbumin was used to induce allergic asthma following administration of YFP for one week in mice, to collect the lung tissues, bronchoalveolar lavage fluid (BLFA), and feces. The pathological state, tight-junction proteins, inflammatory and oxidative stress-associated biomarkers, and TLRs/NF-*κ*B signaling pathway of the lung tissues were evaluated by HE staining, immunofluorescence, ELISA, and WB, separately. RT-PCR was used to test oxidative stress-associated genes. Leukocyte counts of BLFA and intestinal microbiota were also analyzed using a hemocytometer and 16S rDNA-sequencing, separately.

**Result:**

YFP ameliorated the lung injury of the mouse asthma model by inhibiting peribronchial and perivascular infiltrations of eosinophils and increasing tight-junction protein expression. YFP inhibited the decrease in the number of BALF leukocytes and expression of inflammatory-related genes and reversed OVA-induced TLRs/NF-*κ*B signaling pathway activation. YFP ameliorated the level of oxidative stress in the lung of the mouse asthma model by inhibiting MDA and promoting the protein level of GSH-PX, SOD, CAT, and oxidative-related genes. ATG5, Beclin1, and LC3BII/I were significantly upregulated in asthma mice, which were greatly suppressed by the introduction of YFP, indicating that YFP ameliorated the autophagy in the lung of the mouse asthma model. Lastly, the distribution of bacterial species was slightly changed by YFP in asthma mice, with a significant difference in the relative abundance of 6 major bacterial species between the asthma and YFP groups.

**Conclusion:**

Our research showed that YFP might exert antiasthmatic effects by inhibiting airway allergic inflammation and oxidative stress level through suppressing autophagy.

## 1. Introduction

Allergic asthma is a common chronic inflammatory respiratory disease with high morbidity and mortality all over the world. According to the World Health Organization (WHO) report in 2015 [1], approximately 0.3 billion patients are diagnosed with allergic asthma. Allergic asthma is mainly clinically characterized by discontinuous reversible airway obstruction and bronchial hyperresponsiveness [2]. Although numerous investigations have explored allergic asthma's pathogenesis in the past decades, allergic asthma's aetiology and pathogenesis remain unknown, which prevents pharmaceutical companies from developing effective targeted drugs and the clinicians from diagnosing accurately [3].

Airway allergic inflammation is regarded as one of the leading theories on allergic asthma [4]. As allergic asthma develops, large amounts of inflammatory cells infiltrate into the lung tissues in the pathological biopsy of both clinical allergic asthma patients and the experimental animal models, including granulocytes [5], mastocytes [6], macrophages [7], dendritic cells [8], and T cells and B cells [9]. The NF-*κ*B signal pathway is reported to be both involved in inflammatory activation, including TLR4/NF-*κ*B [10], CX3CR1/NF-*κ*B [11], p120/NF-*κ*B [12], and TRAF6/NF-*κ*B [13] signal pathways. Hong et al. [14] reported that bromodomain-containing protein 4 inhibition alleviated matrix degradation by enhancing autophagy and suppressing NLRP3 inflammasome activity through regulating NF-*κ*B signaling in nucleus pulposus cells. Besides, Qi et al. [15] reported that MSTN attenuated cardiac hypertrophy through the inhibition of excessive cardiac autophagy by blocking AMPK/mTOR and miR-128/PPAR*γ*/NF-*κ*B signal pathways. These studies reveal the critical roles of NF-*κ*B for the regulation of inflammations.

Furthermore, the mice with knockout of autophagy gene ATG5 in dendritic cells are more susceptible to sterol-tolerant neutrophilic airway inflammation [16], indicating that autophagy might be involved in the development and progressing of allergic asthma. Autophagy is a relatively conservative degradation of cellular materials, such as damaged organelles or reactive oxygen species (ROS) [17]. Recent studies reveal the high correlation of the nucleotide polymorphism of ATG5/7 and the development of asthma in pediatrics [18] and adults [19]. Besides, more autophagic vacuoles are observed in the clinical-pathological biopsy of allergic asthma patients [20]. Excessive production of ROS induced by the aggravation of autophagy in the tissues further contributes to oxidative stress, while oxidative stress could be suppressed by 3-MA, an autophagy inhibitor, in a murine allergic asthma mouse model [21]. Poon et al. also reported an important role of autophagy-regulated oxidative stress in the developing and processing of asthma [20]. Therefore, investigations on oxidative stress induced by autophagy should help understand allergic asthma's pathogenesis better.

Yeast Fermentate Prebiotics (YFP), a group of live microorganisms, have benefit roles in maintaining the health of the host [22], which could be associated with balancing the microbial community structure, inducing the degradation of antigens [23], and regulating immunity [24]. Recently, YFP was reported to exert therapeutic effects on the treatment of allergic asthma [25–29]. Besides, prebiotic was reported to regulate the NF-*κ*B signal pathway in colitis [30] and diabetes [31] by modulating gut microbiota. In the present study, the antiasthma effects of YFP and the underlying mechanism will be investigated to provide the fundamental basis for the potential therapeutic application of YFP against allergic asthma.

## 2. Materials and Methods

### 2.1. Animals and Allergic Asthma Model

Twenty-four 6-week old BABL/c male mice were purchased from Beijing Vital River Laboratory Animal Technology Co., Ltd. The mice were divided into three groups: the control group, the asthma group, and the asthma+YFP group. The mice in the asthma+YFP group were orally administrated with YFP (1 × 10^9^ CFU/day) from day 0 to day 6, while the other mice received oral administration of normal saline. Ovalbumin (OVA) was used to establish the murine asthma model according to a modification of the methods proposed by Yu et al. [29]. Briefly, the mice in the asthma group and the asthma+YFP group were administrated with an intraperitoneal injection of 20 *μ*g OVA emulsified in 2.25 mg alum hydroxide in a total volume of 100 *μ*L at day 7 and day 14 and inhaled with 1% OVA through an ultrasonic sprayer (Nescosonic UN-511, Alfresa, Osaka, Japan) for three days from day 21. Normal saline was administered orally instead of OVA to the control mice. On day 23, all the mice were sacrificed for the collection of lung and feces. The lungs were flushed twice with cold 0.5% fetal bovine serum in 1 mL PBS, and bronchoalveolar lavage fluid (BALF) was obtained for leukocyte counts using a hemocytometer (Thermo) after lavage and centrifuged at 2000 g at 4°C for 5 min. We declare that all animal experiments involved in this manuscript were authorized by the ethical committee of The Second Xiangya Hospital of Central South University and carried out according to the guidelines for care and use of laboratory animals as well as to the principles of laboratory animal care and protection.

### 2.2. Enzyme-Linked Immunosorbent Assay (ELISA)

Inflammatory or oxidative stress biomarkers, including tumor necrosis factor-*α* (TNF-*α*), interleukin- (IL-) 1*β*, IL-6, transforming growth factor- (TGF-) *β*, interferon- (IFN-) *γ*, malondialdehyde (MDA), glutathione peroxidase (GSH-Px), superoxide dismutase (SOD), and catalase (CAT), in BALF were detected by ELISA according to the instruction of the manufacturer (Sigma-Aldrich, Missouri, USA). The samples were firstly incubated with 1% BSA and then incubated with the primary antibodies for one hour. Subsequently, the samples were mixed with streptavidin-horseradish peroxidase (HRP) conjugated secondary antibodies for 20 mins at room temperature; then, the absorption at 450 nm was analyzed using a microplate spectrophotometer (Thermo Fisher, Massachusetts, USA).

### 2.3. Hematoxylin and Eosin (HE) Staining

The lungs were washed over by sterile water for three hours, dehydrated by 70%, 80%, and 90% ethanol solution successively, and mixed with equal quality of ethanol and xylene. After 15 min incubation, the tissues were mixed with equal quality of xylene for 15 min. Repeat the steps until the tissues looked transparent. Subsequently, the tissues were embedded in paraffin, sectioned, and stained with hematoxylin and eosin (HE). H&E-stained tissue sections were analyzed under a microscope (Olympus).

### 2.4. Real-Time Polymerase Chain Reaction (RT-PCR)

Total RNA of the lungs was extracted using a TaKaRa MiniBEST Universal RNA Extraction Kit (TaKaRa, Dalian, China), according to the manufacturer's instructions, and quantified with a NanoDrop spectrophotometer (NanoDrop Technologies, Wilmington, DE). Complementary DNA was generated with a specific RT primer. RT-PCR was performed with SYBR Premix Ex TaqTM (Tli RNaseH plus) (TaKaRa, Dalian, China) by the Applied Bio-Rad CFX96 Sequence Detection System (Applied Biosystems). The expression level of GPX1-4, CAT, SOD1, SOD2, and UCP2 was defined from the threshold cycle (Ct), and relative expression levels were calculated using the 2^−*ΔΔ*Ct^ method after normalization regarding the expression of U6 small nuclear RNA. The expression level of GAPDH in the tissues was taken as the negative control. Three independent assays were performed. The information of the primers is shown in Table 1.

### 2.5. Immunofluorescence

The BALF cells were incubated with primary rabbit anti-ZO-1, anti-Claudin1, anti-Claudin4, and anti-Occludin (OmnimAbs, 1 : 1000) antibody overnight at 4°C. Following washed three times with PBS, cells were incubated with secondary Cy3-conjugated anti-rabbit IgG (Abcam, 1 : 200) for 30 min at room temperature. The DAPI was added to dye the nuclear for 5 min, and 50% glycerin was used to block the medium. Stained cells were photographed under a fluorescence microscope (Olympus, Tokyo, Japan).

### 2.6. Western Blot

Inflammatory and autophagy-related proteins were evaluated by Western blot. Proteins were extracted from the lung tissues using the Nuclear and Cytoplasmic Protein Extraction Kit (Beyotime, China). Approximately 40 *μ*g of protein was loaded and separated with the 12% SDS-polyacrylamide gel (SDS-PAGE) and then transferred to polyvinylidene difluoride (PVDF) membrane (Millipore, MIT, USA). The membrane was incubated with 5% nonfat dry milk in TBST (Tris-buffered saline/0.1% Tween-20, pH 7.4) for 1 h at room temperature, followed by incubation overnight with primary rabbit anti-mouse antibodies to NF-*κ*B (1 : 1000, Abcam, USA), p-NF-*κ*B (1 : 1000, Abcam, USA), TLR1 (1 : 1000, Abcam, USA), TLR2 (1 : 1000, Abcam, USA), TLR3 (1 : 1000, Abcam, USA), TLR4 (1 : 1000, Abcam, USA), Myd88 (1 : 1000, Abcam, USA), ATG5 (1 : 1000, Abcam, USA), LC3I/II (1 : 1000, Abcam, USA), Beclin1 (1 : 1000, Abcam, USA), and GAPDH (1 : 1000, Abcam, USA). A horseradish peroxidase-conjugated antibody against rabbit IgG (1 : 5000, Abcam, USA) was used as a secondary antibody. Blots were incubated with the ECL reagents (Beyetime, Jiangsu Province, China) and exposed to Tanon 5200-multi to detect protein expression. Three independent assays were performed.

#### 2.6.1. 16S rDNA-Sequencing Analysis

Three feces were collected from each group for the microbiome analysis. Bacterial genomic DNA was extracted from feces using the Qiagen DNA Mini Kit (Qiagen, Valencia, California) according to manufacturer's protocols, and the V4-V5 hypervariable regions of the 16S rDNA gene were PCR-amplified with the appropriate controls against reagent contamination. Amplified DNA fragments were sequenced using the 454 Genome Sequencer FLX platform (454 Life Sciences, Roche Diagnostics, Burgess Hill, United Kingdom). The raw data were processed using quantitative insights into microbial ecology (QIIME) pipeline version 1.7. Stringent criteria were used to remove low quality and chimeric reads. The remaining rarified reads were subject to open reference operational taxonomic unit (OTU) picking (97% identity cutoff). The mean sequencing depth used for analysis was 31615. There is no sample drop-off. The sequence data were deposited to NCBI SRA (Sequence Read Archive) (SRP065072).

### 2.7. Statistical Analysis

GraphPad Prism 7.0 (GraphPad Software, USA) was employed to perform statistical analysis. Results were statistically analyzed with Student's *t*-test for two-group comparisons. Data are presented as mean ± SEM. *P* values < 0.05 were considered significant.

## 3. Results

### 3.1. YFP Ameliorated the Lung Injury of the Mouse Asthma Model

**Figure 1:**
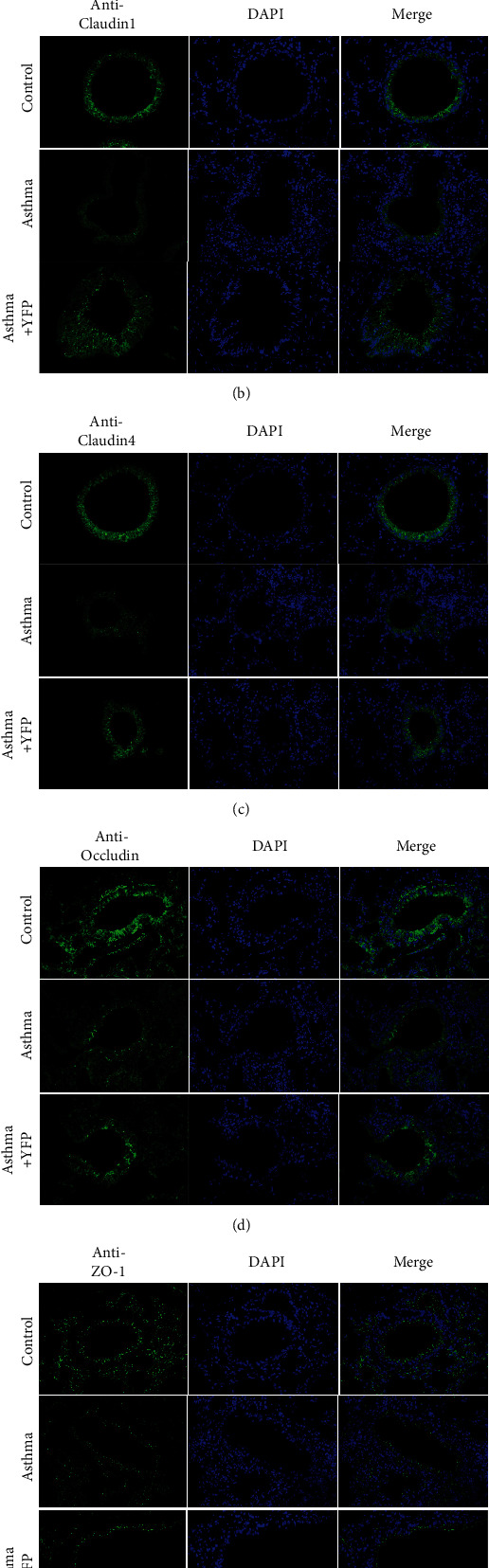
YFP ameliorated the injury of lung tissue of the mouse asthma model. HE staining of the lung tissue from each mouse (a). The expression of Claudin1 (b), Claudin4 (c), Occludin (d), and ZO-1 (e) was detected by immunofluorescence.

HE staining was used to check the pathological state of the lung tissues, and the immunofluorescence assay was used to determine the expression level of tight junction-related proteins. As shown in Figure 1(a), significant peribronchial and perivascular infiltrations of eosinophils were observed in the asthma group, compared to the control, which were significantly suppressed by YFP. ZO-1, Claudin1, Claudin4, and Occludin were significantly downregulated in the asthma group, compared to the control, the expression of which was significantly promoted by the treatment of YFP (Figures 1(b)–1(d)).

### 3.2. YFP Inhibited the Inflammation in the Lung of the Mouse Asthma Model

**Figure 2:**
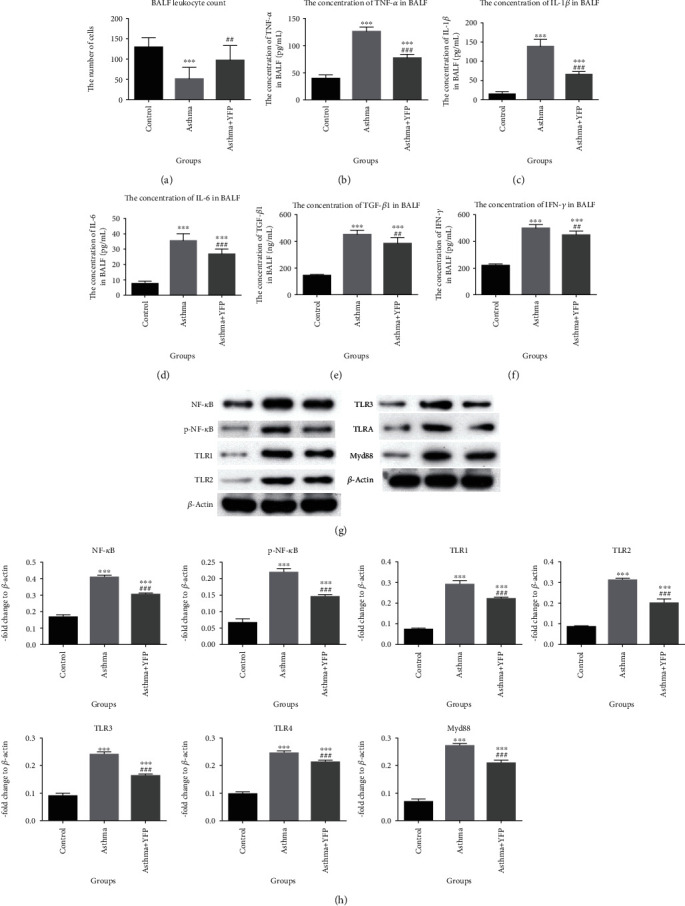
YFP promoted the inflammation in the lung of the mouse asthma model. (a) The BALF leukocyte count was checked by a hemocytometer. (b–f) The expression of TNF-*α*, IL-1*β*, IL-6, TGF-*β*, and IFN-*γ* was determined by ELISA. (g, h) The expression of NF-*κ*B, p-NF-*κ*B, TLR1, TLR2, TLR3, TLR4, and Myd88 was evaluated by Western blot. Data are presented as mean ± SEM. ^∗∗∗^*P* < 0.001 vs. control, ^##^*P* < 0.01 vs. asthma, and ^###^*P* < 0.001 vs. asthma.

YFP inhibited the decrease in the number of BALF leukocytes induced by OVA (Figure 2(a)). As shown in Figures 2(b)–2(f), the concentration of TNF-*α*, IL-1*β*, IL-6, TGF-*β*, and IFN-*γ* was significantly elevated in the asthma group compared to the control group, which was suppressed by the introduction of YFP. To further evaluate the effects of YFP on the inflammation, the expression of related proteins in the lung tissue was detected by Western blot. As shown in Figures 2(g) and 2(h), the expression level of NF-*κ*B, p-NF-*κ*B, TLR1, TLR, TLR3, TLR4, and Myd88 was significantly promoted in the asthma group, compared with the control group, which was also inhibited by the treatment with YFP (^∗∗^*P* < 0.01 vs. control, ^∗∗∗^*P* < 0.001 vs. control, and ^###^*P* < 0.001 vs. asthma).

### 3.3. YFP Ameliorated the Level of Oxidative Stress in the Lung of the Mouse Asthma Model

**Figure 3:**
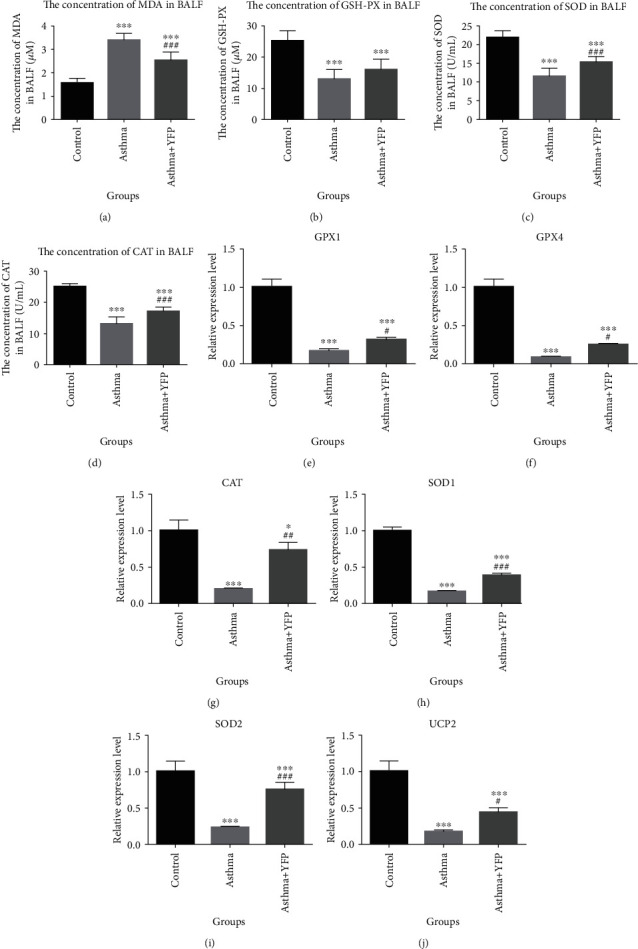
YFP ameliorated the level of oxidative stress in the lung of the mouse asthma model. (a–d) The concentration of MDA, GSH-Px, SOD, and CAT was determined by ELISA. (e–j) The gene expression level of GPX1, GPX4, CAT, SOD1, SOD2, and UCP2 was detected by qRT-PCR. Data are presented as mean ± SEM. ^∗^*P* < 0.05 vs. control, ^∗∗∗^*P* < 0.001 vs. control, ^#^*P* < 0.05 vs. asthma, ^##^*P* < 0.01 vs. asthma, and ^###^*P* < 0.001 vs. asthma.

Oxidative stress-related factors were detected in the BALF by ELISA to evaluate the effect of YFP on oxidative stress. Compared to the controls, MDA was significantly improved in the asthma group, which was inhibited by the introduction of YFP (Figure 3(a)), while the decreased production of GSH-PX, SOD, and CAT in the asthma group was significantly elevated by the treatment of YFP (Figures 3(b)–3(d)). Besides, the expression of oxidative stress-related genes in the lung tissue was detected by qRT-PCR. The results are shown in Figures 3(e)–3(j). The gene expression level of GPX1, GPX4, CAT, SOD1, SOD2 and UCP2 was significantly suppressed in the asthma group, compared to the control group, and was promoted comparing with that by the asthma+YFP group (^∗∗^*P* < 0.01 vs. control, ^∗∗∗^*P* < 0.001 vs. control, and ^###^*P* < 0.001 vs. asthma).

### 3.4. YFP Ameliorated the Autophagy in the Lung of the Mouse Asthma Model

**Figure 4:**
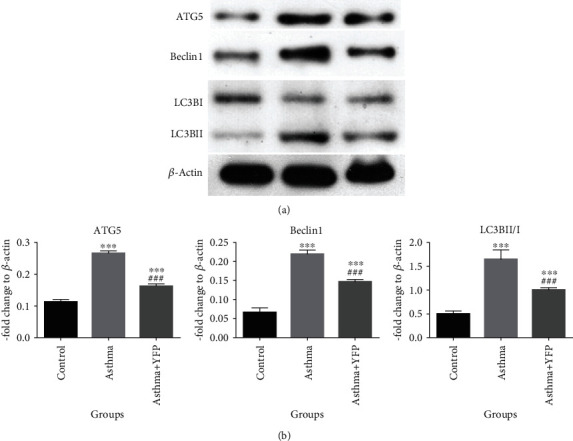
YFP ameliorated the level of oxidative stress in the lung of the mouse asthma model. (a) The expression level of ATG5, Beclin1, and LC3I/II detected by Western blot. (b) The quantitative results of the protein expression. Data are presented as mean ± SEM. ^∗∗^*P* < 0.01 vs. control, ^∗∗∗^*P* < 0.001 vs. control, and ^###^*P* < 0.001 vs. asthma.

To evaluate the effects of YFP on autophagy in the lung tissues induced by asthma modeling, Western blot was used to determine the expression level of autophagy-related proteins. As shown in Figure 4, ATG5, Beclin1, and LC3BII/I were significantly upregulated in asthma mice compared to control, which were greatly suppressed by the introduction of YFP (^∗∗^*P* < 0.01 vs. control, ^∗∗∗^*P* < 0.001 vs. control, and ^###^*P* < 0.001 vs. asthma).

### 3.5. The Distribution of Bacteria Species Was Slightly Changed by YFP in Asthma Mice

**Figure 5:**
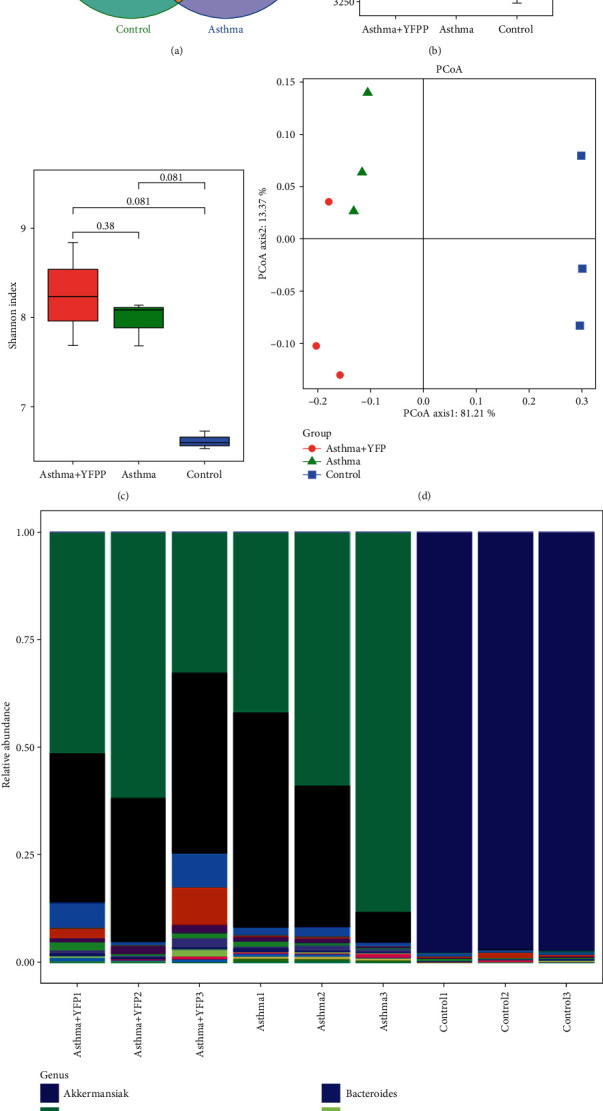
The distribution of gut microbiota was regulated by YFP. (a) Venn diagram of shared genus observed in the feces samples. (b) The microbial richness (Chao1) analysis in each group. (c) Alpha diversity (Shannon) analysis in each group. (d) The PCoA analysis in each group. (e) The histogram of species distribution of the top 15 with the highest relative abundance.

16S rDNA sequencing analysis was performed to explore the effect of YFP on the gut microbiota of allergic asthma mice. As shown in Figure 5(a), the Venn diagram revealed that a total of 1045 distinct genera were identified upon YFP treatment. These observations indicated that the YFP treatment increased the bacterial diversity in the gut of the animal, although not statistically significant. Figures 5(b)–5(d) showed the microbial richness (Chao1) analysis, alpha diversity (Shannon) analysis, and PCoA analysis, respectively. However, no significant difference was observed between the asthma group and the YFP-treated group. As shown in Figure 5(e) and Table 2, the major difference in the relative abundance of the bacteria species in the feces of mice was observed between the control group and the asthma group, of which the top 15 species with the highest abundance were listed. Interestingly, except for *Akkermansia* (higher in the control group), the relative abundance of *Prevotella*, *Oscillospira*, *Helicobacter*, *Coprococcus, Ruminococcus*, *Bacteroides*, *Flexispira*, *Odoribacter*, and *Turicibacter* in the asthma mice was significantly elevated, compared to control (^∗^*P* < 0.05 vs. control). By the treatment of YFP, the relative abundance of *Oscillospira*, *Helicobacter*, *Coprococcus*, *Ruminococcus*, *Flexispira*, and *Odoribacter* was greatly decreased (^#^*P* < 0.05 vs. asthma).

## 4. Discussion

Airway allergic inflammation is reported to be one of the pathological basis of allergic asthma [32]. Large amounts of inflammatory immune cells are recruited into the lung tissues when allergic occurs and release inflammatory cytokines to accelerate airway allergic inflammation, promoting aggravate allergic asthma process [33]. Therefore, it is of great importance to inhibiting airway inflammation in allergic asthma. In the present study, the antiasthma effect of YFP was investigated. HE staining showed that YFP ameliorated the lung injury caused by asthma modeling in mice. The tight-junction protein of lung tissues is closely related to the pathological state of allergic asthma, which is represented by the expression level of ZO-1, Claudin1, Claudin4, and Occludin [34, 35]. After the administration of YFP, the tight junction of lung tissue was greatly improved. The results indicated that the symptom of allergic asthma in mice was significantly improved by the administration of YFP. Besides, YFP promoted inflammation in the lung. By exploring the state of the inflammatory signal pathways in the lung tissues following treatments with YFP, we found that YFP greatly inhibited the TLR/NF-*κ*B signal pathway in the lung tissue. These data implied that the symptom of allergic asthma in the mouse model had been greatly improved by YFP, accompanied by suppression of the airway allergic inflammation.

To further investigate the possible mechanism underlying the inflammation inhibitory effects of YFP, the distribution of gut microbiota in the feces of mice was explored. Salameh et al. [36] reported that gut microbiota plays a great role in the pathogenesis of allergic asthma, to which the inflammatory factors might be the mediators. In the present study, we found that although the relative abundance of the top 3 bacteria species was not reversed by YFP in the asthma mice, a significant inhibitory effect on the relative abundance of the top 4-8 bacteria species was achieved by the treatment of YFP, indicating a minor impact of YFP on the distribution of gut microbiota of the asthma mice. However, more evidence of the positive correlation between the antiasthma property of YFP and the change of gut microbiota distribution should be provided in our future work. In the present study, only one dosage of YFP was applied and we suspected that the change of gut microbiota distribution, such as the relative abundance of the bacteria species, *α*-diversity index, and *β*-diversity index, might be enlarged if we increased the dosage, which will be verified in our subsequent work. Besides, in our future work, the inflammation profile both in the central nervous system and in the peripheral region will also be described to analyze the change of inflammation state before and after the treatment of YFP, which might bring direct evidence to explain the antiasthma property of YFP.

Oxidative stress is also a pathological manifestation of allergic asthma, the representative indexes of which are MDA, GSH-PX, SOD, and CAT [37]. In the present study, the oxidative stress level in the asthmatic mice was significantly inhibited by the treatment of YFP. Silveira et al. [21] reported that autophagy could induce eosinophil extracellular trap formation and allergic airway inflammation in a murine asthma model by activating oxidative stress. Tsai et al. [38] also reported that autophagy against oxidative stress-mediated apoptosis in normal and asthmatic airway epithelium is induced by complement regulatory protein CD46. In the present study, the expression level of autophagy-related proteins (ATG5, Beclin1, and LC3BII/I) was significantly suppressed by YFP. These data indicated that YFP might exert antiasthmatic effects by inhibiting oxidative stress through suppressing autophagy.

According to the data achieved in the present study, on the one hand, the inflammation induced in the murine asthma model could be alleviated by the treatment of YFP, which might be related to the regulatory effect of YFP on the TLR4/NF-*κ*B signaling pathway. In our future work, the specific inhibitor against the TLR4/NF-*κ*B signaling pathway will be introduced to verify the mechanism underlying the inhibitory effect of YFP against inflammation. On the other hand, oxidative stress in the lung tissues was ameliorated by YFP by regulating autophagy, which should also be verified by introducing the agonist of autophagy in our future work. As a result, the symptom of asthma in the murine model was significantly alleviated.

Collectively, our research showed that YFP might exert antiasthmatic effects by inhibiting airway allergic inflammation through regulating gut microbiota distribution and inhibiting oxidative stress levels through suppressing autophagy.

## Figures and Tables

**Table 1:** The primers used in this study.

Primer name	Primer sequence (5′-3′)
GPX1 forward	CAGTTGCAGTGCTGCTGTCTC
GPX1 reverse	GCTGACACCCGGCACTTTATTAG
GPX2 forward	GACACGAGGAAACCGAAGCA
GPX2 reverse	GGCCCT TCACAACGTCT
GPX3 forward	CTTCCTA CCCTCAAGTATGTCCG
GPX3 reverse	GAGGTGGGAGGACAGGAGTT CTT
GPX4 forward	GCAACCAGTTTGGGAGGCAGGAG
GPX4 reverse	CCTCCATGGGACCATAGCGC TTC
CAT forward	GGTCATGCATTTAATCAGGCAGAA
CAT reverse	TTGCTTGGGTCGAAGGCTATC
SOD1 forward	GAAGGTGTGGGGAAGCATTA
SOD1 reverse	ACATTGCCCAAGTCTCCAAC
SOD2 forward	CCAAATCAGGATCCACTGCAA
SOD2 reverse	CAGCATAACGATCGTGGTTTACTT
UCP2 forward	CTACAAGACCATTGCACGAGAGG
UCP2 reverse	AGCTGCTCATAGGTGACAAACAT
GAPDH forward	CAATGACCCCTTCATTGACC
GAPDH reverse	GAGAAGCTTCCCGTTCTCAG

**Table 2:** The relative abundance of the top 15 bacteria species in the control, asthma, and YFP groups (^∗^*P* < 0.05 vs. control, ^#^*P* < 0.05 vs. asthma).

Name of genus	Relative abundance in the control group	Relative abundance in the asthma group	Relative abundance in the YFP group
Akkermansiak	0.376830 ± 0.018474	4.042394 ± 1.664278^∗^	7.308090 ± 3.401218
Prevotella	0.000692 ± 0.000574	0.151322 ± 0.065380^∗^	0.153308 ± 0.083194
[Prevotella]	7.146626 ± 1.974750	0.108791 ± 0.008467^∗^	0.066855 ± 0.048877
Oscillospira	0.002609 ± 0.000602	0.013902 ± 0.009423^∗^	0.003553 ± 0.001185^#^
Helicobacterk	0.002436 ± 0.001818	0.010009 ± 0.010251^∗^	0.000643 ± 0.000375^#^
Coprococcus	0.000674 ± 0.000434	0.004445 ± 0.001347^∗^	0.001724 ± 0.000896^#^
Ruminococcus	0.001198 ± 0.000623	0.003315 ± 0.002597^∗^	0.001678 ± 0.001107^#^
[Ruminococcus]	0.000406 ± 9.398833	0.003168 ± 0.001985^∗^	0.001569 ± 0.000606^#^
Bacteroides	0.000609 ± 0.000204	0.001355 ± 0.000624^∗^	0.001191 ± 0.000658
Flexispira	0.000674 ± 0.000652	0.001800 ± 0.002127^∗^	0.000515 ± 0.000160^#^
Parabacteroides	0.000714 ± 0.000509	0.000943 ± 0.000621	0.001299 ± 0.001236
Odoribacter	0 ± 0	0.001427 ± 0.000976^∗^	0.000848 ± 0.000512^#^
Sutterella	0.000279 ± 0.000132	0.000289 ± 0.000168	0.001239 ± 0.000139
Turicibacter	0 ± 0	0.000369 ± 0.000155^∗^	0.001182 ± 0.000549^#^
AF12	0.000279 ± 0.000139	0.000472 ± 0.000161	0.000693 ± 0.000180

## Data Availability

The data can be available if requested by the editor.
